# Supplementary material for: Ionizing Radiation Selectively Increases CXC Ligand 10 Level via the DNA-Damage-Induced p38 MAPK-STAT1 Pathway in Murine J774A.1 Macrophages
Source: Cells. 2023 Mar 25;12(7):1009. doi: 10.3390/cells12071009 (PMC10093567; doi:10.3390/cells12071009)
Supplement: Supplementary file 1 [file cells-12-01009-s001.zip › cells-2221122-supplementary.pdf]

Supplementary Table S1. Primers used for PCR analysis in this study

| Gene | | Sequence (5’ → 3’) | Tm(℃) |
| --- | --- | --- | --- |
| *Cxcl10* | F | 5’-CCAAGTGCTGCCGTCATTTT-3’ | 57.5 |
| | R | 5’-CTCAACACGTGGGCAGGATA-3’ | 58.4 |
| *Stat1* | F | 5’-ACAGTTCACTGTCAAGTCGAG-3’ | 55.3 |
| | R | 5’-CGTTGGAGATCACCACGACA-3’ | 58.5 |
| *Gapdh* | F | 5’-CTGTGGATGGCCCCTCTGGA-3’ | 58.7 |
| | R | 5’-GGAAGGCCATGCCCAGTGAGC-3’ | 55.3 |

Supplementary Table S2. The company, catalog number, and dilution of all antibodies used in this study

| **Antibody** | **Company** | **Catalog No.** | **Dilution rate** |
| --- | --- | --- | --- |
| p-ATM | Cell Signaling Technology | #5883 | 1:1000 |
| ATM | Cell Signaling Technology | #2873 | 1:1000 |
| p-CHK1 | Cell Signaling Technology | #2348 | 1:1000 |
| CHK1 | Cell Signaling Technology | #2360 | 1:1000 |
| γ-H2AX | Cell Signaling Technology | #9718 | 1:1000 |
| H2AX | Cell Signaling Technology | #7631 | 1:1000 |
| p-STAT1 | Cell Signaling Technology | #9167 | 1:1000 |
| STAT1 | Cell Signaling Technology | #9172 | 1:1000 |
| p-AKT | Cell Signaling Technology | #4060 | 1:1000 |
| AKT | Cell Signaling Technology | #9272 | 1:1000 |
| p-JNK | Cell Signaling Technology | #9255 | 1:1000 |
| JNK | Cell Signaling Technology | #9252 | 1:1000 |
| p-P38 | Cell Signaling Technology | #9215 | 1:1000 |
| P38 | Cell Signaling Technology | #9212 | 1:1000 |
| p-ERK | Cell Signaling Technology | #4370 | 1:1000 |
| ERK | Cell Signaling Technology | #9102 | 1:1000 |
| ACTIN | Sigma-Aldrich | A5441 | 1:1000 |
| Anti-Rabbit | Invitrogen | G-21234 | 1:5000 |
| Anti-Mouse | Invitrogen | G-21040 | 1:5000 |

Supplementary Figure S1

A

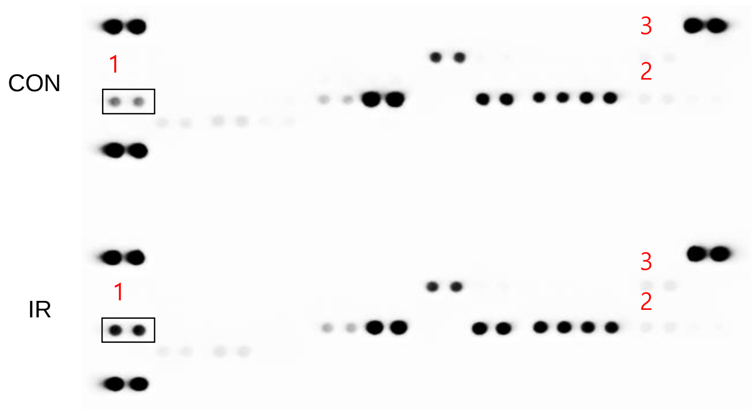


B

| cytokines | Fold change | P-value |
|---|---|---|
| CXCL10 | 10.68 ± 0.0080 | <0.05 |
| CCL5 | 6.87 ± 0.0019 | 0.098 |
| IL-1ra | 5.15 ± 0.0009 | <0.05 |
| CCL12 | 1.83 ± 0.0009 | <0.001 |
| CCL3 | 1.34 ± 0.0027 | <0.01 |
| CD54 | 1.31 ± 0.0023 | <0.01 |
| TNF-a | 1.29 ± 0.0001 | <0.01 |
| CCL4 | 1.18 ± 0.0026 | <0.05 |
| CCL2 | 1.07 ± 0.0020 | 0.114 |
| CXCL2 | 0.98 ± 0.0068 | 0.664 |
| TIMP-1 | 0.9 ± 0.0004 | 0.353 |
| TREM-1 | undetected | NA |
| CXCL13 | undetected | NA |
| C5/C5a | undetected | NA |
| G-CSF | undetected | NA |
| GM-CSF | undetected | NA |
| I-309 | undetected | NA |
| CCL11 | undetected | NA |
| CD54 | undetected | NA |
| IFN-γ | undetected | NA |
| IL-1α | undetected | NA |
| IL-1β | undetected | NA |
| IL-2 | undetected | NA |
| IL-3 | undetected | NA |
| IL-4 | undetected | NA |
| IL-5 | undetected | NA |
| IL-6 | undetected | NA |
| IL-7 | undetected | NA |
| IL-13 | undetected | NA |
| IL-12p70 | undetected | NA |
| IL-16 | undetected | NA |
| IL-17 | undetected | NA |
| IL-23 | undetected | NA |
| IL-27 | undetected | NA |
| CXCL11 | undetected | NA |
| CXCL1 | undetected | NA |
| M-CSF | undetected | NA |
| CXCL9 | undetected | NA |
| CXCL12 | undetected | NA |
| CCL17 | undetected | NA |

C

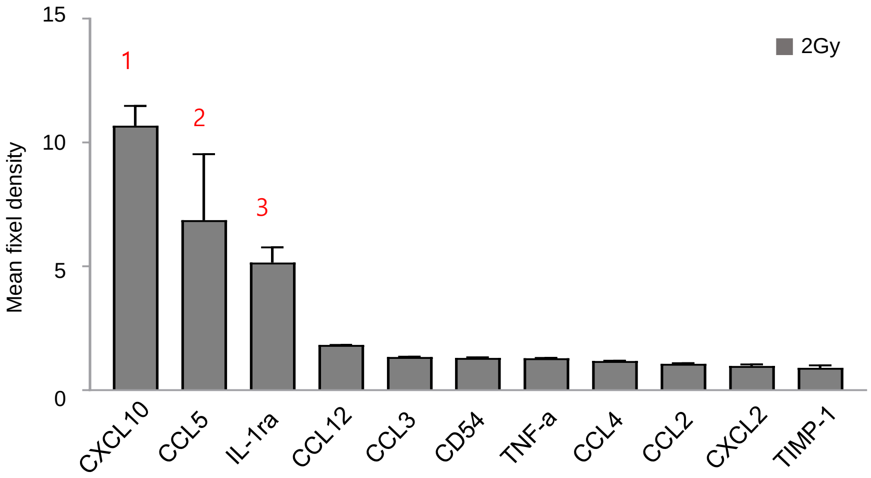

Supplementary Figure S1

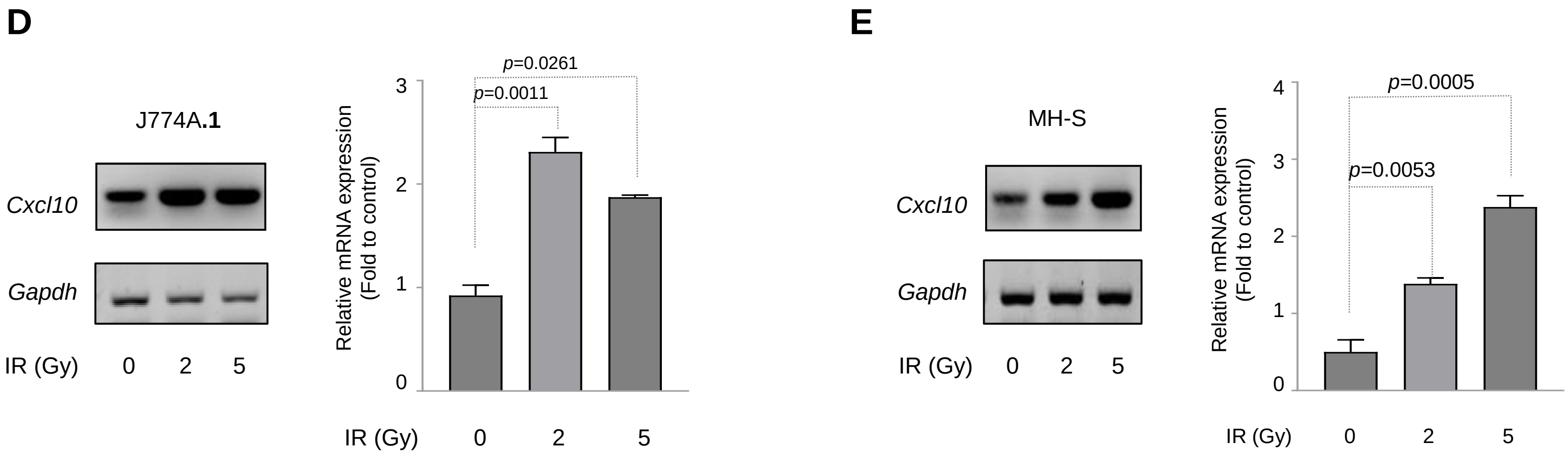


**Figure S1**: Results of cytokine array in irradiated J774A.1 macrophages and changes in CXCL10 mRNA expression by IR in J774A.1 and MH-S macrophage cells.

**A**: Irradiated J774A.1 macrophages and the control J774A.1 macrophages.

**B**: The total cytokine array results.

**C**. The secretion of various cytokines.

**D**. Changes in CXCL10 mRNA expression by IR in J774A.1 macrophage cells.

E. Changes in CXCL10 mRNA expression by IR in MH-S macrophage cells.

Supplementary Figure S2. Changes in STAT1 mRNA expression by IR in J774A.1 and MH-S macrophage cells

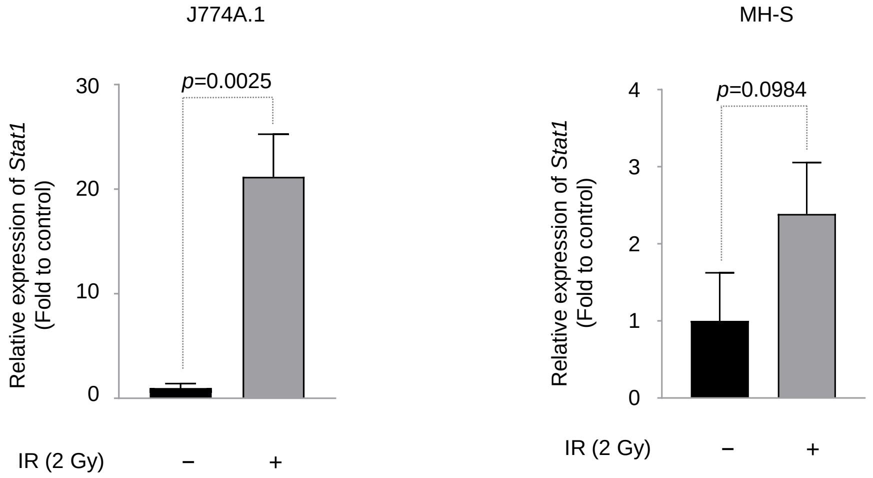

Supplementary Figure S3

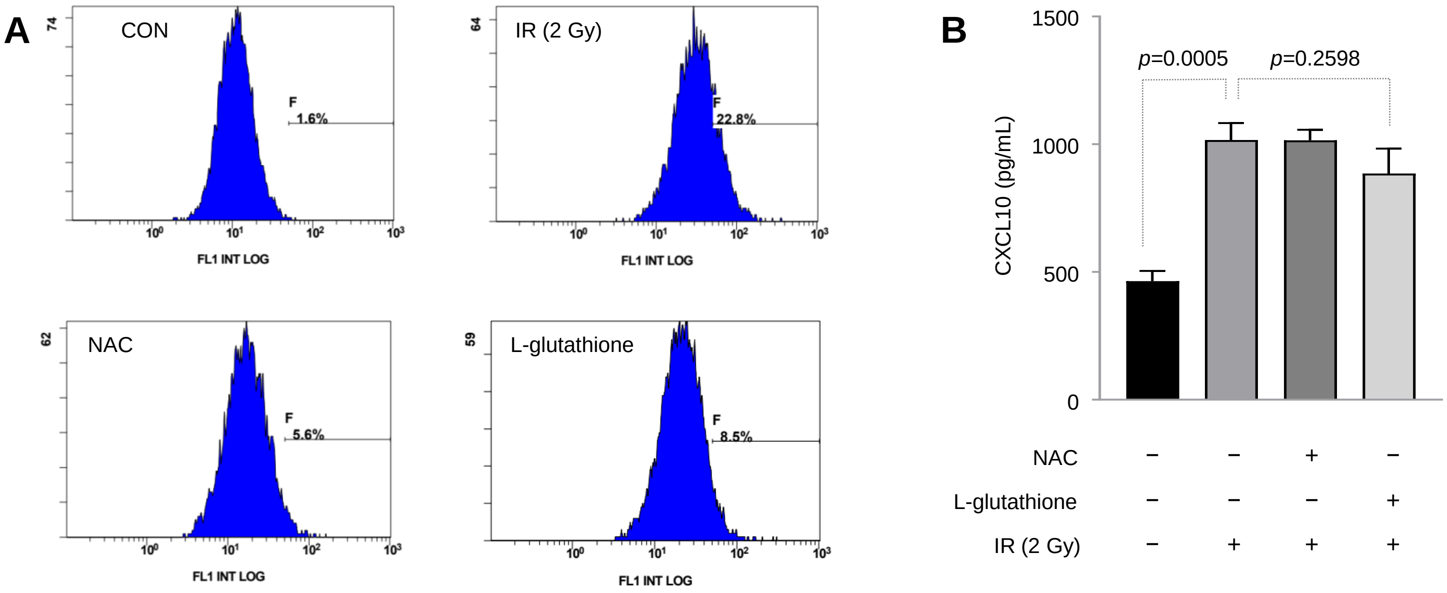


Reactive oxygen species (ROS) production by ionizing radiation is not involved in the production of CXCL10 in irradiated J774A.1 macrophages. 2 × $10^5$ cells/mL of the J774A.1 macrophage cell line, with or without the ROS scavengers N-acetyl-cysteine (NAC) and glutathione, was exposed to 2 Gy of radiation, and 48 hours later, (**A**) the degree of ROS generation by the cells and (**B**) the amount of CXCL10 released into the cell culture medium was measured. NAC (0.2 mM) and glutathione (30 μM) were pretreated 30 minutes before irradiation. (**A**) Ionizing radiation increased the production of ROS in J774A.1 macrophages. In addition, the well-known ROS scavengers NAC and glutathione suppressed the generation of ROS by ionizing radiation. (**B**) On the contrary, NAC and glutathione did not inhibit the production of CXCL10 by ionizing radiation (n = 3). Bar graphs are presented as the mean ± standard error of the mean value.

*Effect of increased ROS production by IR on CXCL10 production in J774A.1 macrophages*

A significant biological effect of IR on cells is ROS generation. We investigated whether IR increased ROS production in the J774A.1 macrophage cell line under our experimental conditions as well as how increased CXCL10 production by IR changes when ROS production is inhibited with a pharmacological inhibitor. Supplementary Figure 3A shows that ROS production increased from 1.6% to 22.8% by 2 Gy IR in J774A.1 macrophages. In addition, two pharmacological inhibitors (0.2 mM of N-acetyl-cysteine [NAC] and 30 μM of L-glutathione) were used as IR-induced ROS generation inhibitors, both of which reduced IR-induced ROS generation by 5.6% and 8.5%, respectively. In addition to measuring ROS, an experiment was conducted to measure CXCL10 secretion using the supernatant of J774A.1 macrophages. In contrast to the results of the ROS measurements, neither drug used as a ROS production inhibitor blocked the IR-induced increase in CXCL10 production in the J774A.1 macrophage cell line (Supplementary Figure 3B). These results suggest that increased ROS production by IR in J774A.1 macrophages is not directly related to increased CXCL10 secretion.

Supplementary Figure S4

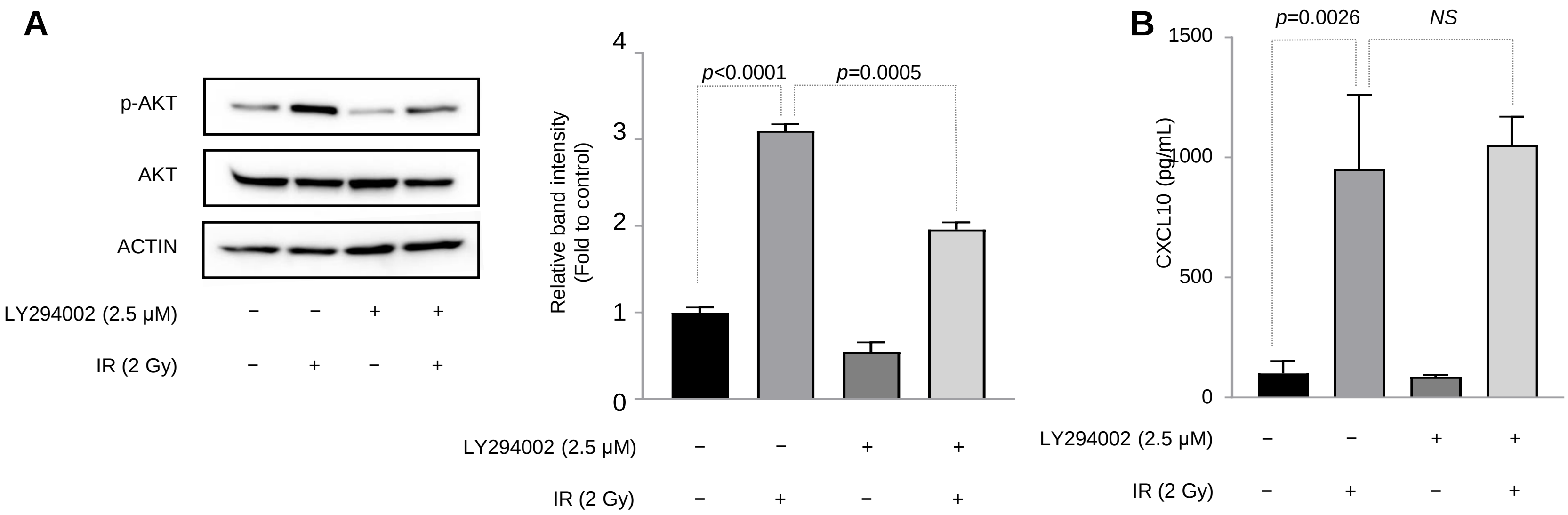


PI3K-Akt pathway activation by ionizing radiation is not involved in the production of CXCL10 in irradiated J774A.1 macrophages. $2 \times 10^5$ cells/mL of the J774A.1 macrophage cell line, with or without PI3K inhibitor LY294002 (2.5 μM) was exposed to 2 Gy of radiation, and 48 hours later, (A) the total Akt and phosphorylated Akt protein expression and (B) the amount of CXCL10 released into the cell culture medium was measured. LY294002 was pretreated 30 minutes before irradiation (n = 3). Bar graphs are presented as the mean ± standard error of the mean value.

*The PI3K-Akt signaling pathway is not involved in the IR-induced increase in CXCL10 production in J774A.1 macrophages*

To identify the upstream target of STAT1, the PI3K-Akt signaling pathway was first identified. IR increased the Akt protein's phosphorylation in J774A.1 macrophages (Supplementary Figure 4A). In addition, this IR-induced increase in phosphorylation was inhibited by the PI3K inhibitor LY294002 (2.5 μM). In contrast, LY294002 did not inhibit the increase in CXCL10 production by IR (Supplementary Figure 4B). These results suggest that although IR increases Akt protein phosphorylation in J774A.1 macrophages, it is not involved in the increase in CXCL10 production by IR.
